# Should they stay or should they go? Regulating microRNA turnover in plants

**DOI:** 10.1093/plcell/koag077

**Published:** 2026-03-16

**Authors:** Soledad Traubenik

**Affiliations:** Assistant Features Editor, the Plant Cell, American Society of Plant Biologists; Institute of Plant Sciences Paris-Saclay (IPS2), CNRS, INRAE, Université Paris-Cité, Gif-sur-Yvette 91190, France

MicroRNAs (miRNAs) operate at the core of gene regulation in plants, guiding development and helping plants respond to a constantly changing environment. Fluctuations in temperature, nutrient availability, and stress conditions often require rapid and precise adjustments in gene expression (Manavella et al. 2019). Because miRNAs' regulatory impact strongly depends on their abundance, plants must carefully maintain or actively adjust miRNA dosage in a spatially and temporally controlled manner (Wang et al. 2019). We often think of their abundance as something largely fixed by biogenesis: once produced, miRNAs accumulate and do their job, but this view misses an important side of the story; like any regulatory molecule, miRNAs must also be removed. Much of what we know about plant miRNAs comes from studies of their biogenesis and mode of action (Rogers et al. 2013), but what happens to miRNAs once they are active has received far less attention. Are they simply stable end products, or are they actively turned over? We know that miRNA decay is regulated and biologically meaningful, but the global view of miRNA stability and the mechanisms controlling it remain unclear.

To tackle this gap, Fang and colleagues (2026) asked how long individual miRNAs persist in the cell. Using cordycepin (3′-deoxyadenosine), a nucleoside analog that inhibits RNA synthesis, the authors implemented a transcriptional shut-off approach to measure miRNA turnover in *Arabidopsis*. This strategy allowed them to build a comprehensive dataset of miRNA stability across tissues and conditions. While a large fraction of miRNAs were remarkably stable, another subset displayed rapid turnover, with considerably shorter half-lives. Importantly, this heterogeneity was not random: passenger strands were overrepresented among fast-decaying species, turning over significantly faster than their corresponding guide strands. But this was not a strict rule; the subset of miRNAs that were more frequently enriched in AGO complexes displayed notable stability, suggesting that AGO association, rather than strand identity/sequence, contributes to miRNA protection.

But if protection is a major determinant of stability, what happens when that protection is compromised? Examining mutants defective in AGO1 and in HEN1-mediated 3′ methylation (a modification that promotes the stability of miRNAs), the authors showed that miRNA decay was accelerated, with short-lived species becoming more unstable. Even typically long-lived miRNAs showed increased turnover when these protective pathways were disrupted. These results reinforced the idea that miRNA lifespan is not an intrinsic property of the molecule alone, but rather the outcome of an active stabilization mechanism. Beyond protection, Fang and colleagues identified an active driver of selective miRNA decay: the F-box protein HAWAIIAN SKIRT (HWS) (Gonzalez-Carranza et al. 2007) emerged as a crucial regulator of short-lived guide miRNAs. Loss of HWS stabilized normally unstable miRNAs, whereas its overexpression accelerated their decay, indicating that miRNA degradation is actively controlled rather than being a passive turnover mechanism.

Through genetic and molecular analyses, the authors showed that HWS selectively promotes miRNA decay, establishing it as a key component of the machinery that fine-tunes miRNA dosage in response to developmental and environmental cues (see Fig. 1). Notably, HWS does not primarily target passenger strands. Instead, it appears to act selectively on specific miRNA species. Recent work has implicated an antagonistic role of HWS in controlling miRNA biogenesis and movement through its interaction with HASTY at the nuclear pore complex, suggesting that HWS may orchestrate miRNA abundance at multiple regulatory levels rather than acting solely at the decay step (Gonzalo et al. 2025). Importantly, HWS-mediated turnover has clear physiological consequences. During oxidative stress, rapid post-transcriptional reduction of miR398 facilitates the induction of antioxidant genes, and under phosphate fluctuations, HWS contributes to miR399 homeostasis. These examples demonstrate that selective miRNA decay is not merely a quality-control mechanism but a dynamic regulatory node integrating environmental signals.

**Figure 1:**
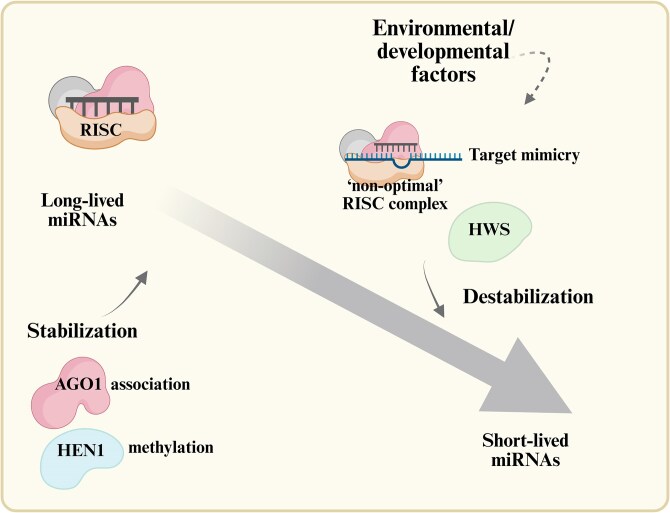
Plant miRNA stability and turnover. Most mature miRNAs are long-lived, largely due to stabilization by AGO1 association and HEN1-mediated 3′ methylation. However, a subset of miRNAs exhibits reduced stability and can undergo selective turnover promoted by the F-box protein HWS. In specific contexts, target mimic RNAs can promote the formation of non-optimal RISC complexes that may facilitate HWS-dependent miRNA destabilization. This selective mechanism contributes to context-dependent modulation of miRNA output. Created using BioRender.

By revealing regulated turnover as an integral part of miRNA homeostasis, Fang and colleagues add a new layer to our understanding of miRNA regulation in plants; deciding whether a miRNA stays or goes is not a passive outcome but an active choice that helps plants adjust gene regulation to a changing environment.

## Recent related articles in *The Plant Cell*:

Cao et al. 2025 uncovered how heat stress promotes miRNA production via regulated HYL1 nuclear localization, enhancing plant thermotolerance.

Pavan et al. 2025 dissected the evolutionary maturation of newly emerged plant microRNAs into canonical regulatory elements.

Genschik et al. 2024 discussed how proteolysis regulates key components of the RNA silencing machinery, highlighting how protein turnover modulates small-RNA pathways.

## Data Availability

No datasets were generated or analysed.
